# Total ankle replacement design and positioning affect implant-bone micromotion and bone strains

**DOI:** 10.1016/j.medengphy.2017.01.022

**Published:** 2017-04

**Authors:** Ran S. Sopher, Andrew A. Amis, James D. Calder, Jonathan R.T. Jeffers

**Affiliations:** a Department of Mechanical Engineering, Imperial College London, 715 City & Guilds Building, South Kensington, London SW7 2AZ, UK; b Department of Surgery & Cancer, Imperial College London, Charing Cross Hospital, London, W6 8RP, UK; c Fortius Clinic, 17 Fitzhardinge St, London, W1H 6EQ , UK

**Keywords:** Total ankle replacement, Fixation, Micromotion, Malpositioning, Finite element modelling

## Abstract

Implant loosening – commonly linked with elevated initial micromotion – is the primary indication for total ankle replacement (TAR) revision. Finite element modelling has not been used to assess micromotion of TAR implants; additionally, the biomechanical consequences of TAR malpositioning – previously linked with higher failure rates – remain unexplored. The aim of this study was to estimate implant-bone micromotion and peri-implant bone strains for optimally positioned and malpositioned TAR prostheses, and thereby identify fixation features and malpositioning scenarios increasing the risk of loosening. Finite element models simulating three of the most commonly used TAR devices (BOX^®^, Mobility^®^ and Salto^®^) implanted into the tibia/talus and subjected to physiological loads were developed. Mobility and Salto demonstrated the largest micromotion of all tibial and talar components, respectively. Any malpositioning of the implant creating a gap between it and the bone resulted in a considerable increase in micromotion and bone strains. It was concluded that better primary stability can be achieved through fixation nearer to the joint line and/or while relying on more than a single peg. Incomplete seating on the bone may result in considerably elevated implant-bone micromotion and bone strains, thereby increasing the risk for TAR failure.

## Highlights

• A finite element model was developed to calculate micromotion of ankle implants.
• Both optimally-positioned and malpositioned cases were considered.
• Fixation nearer to the joint line relying on plural pegs improved implant stability.
• Gaps between the implant and bone greatly increased micromotion and bone strains.

## 1 Introduction

Total ankle replacement (TAR) can provide arthritis patients with pain relief and improved ankle range of motion, and is therefore gaining popularity as an alternative to arthrodesis [1], [2]. The currently used semi-constrained cementless designs with mobile-bearing polyethylene (PE) insert have shown promising results [2].

Loosening of the tibial or talar component is the primary indication for TAR revision (19–47%, [3], [4], [5], [6], [7]). High levels of micromotion of cementless orthopaedic prostheses (>50–150 µm; [8], [9], [10]) are thought to impede osseointegration at the bone-implant interface, thereby hampering fixation [11] and potentially leading to clinical loosening [8], [9], [12]. Accordingly, micromotion of two TAR prosthesis designs has been assessed experimentally to evaluate the implant primary stability using optical tracking [13].

A useful tool to assess initial micromotion of joint replacement implants and peri-implant bone strains is finite element modelling (FEM) (e.g. hip, [14], [15]; shoulder, [16], [17], [18]). Several studies have employed FEM to explore the performance of current TAR devices: Terrier et al. [19], [20], [21] modelled the Salto^®^ implanted in the tibia to explore bone strains and stresses occurring at the implant vicinity. Espinosa et al. [22] developed a model to study contact pressures occurring in the PE component of the Agility^®^ and Mobility^®^. Reggiani et al. [23] included ligaments in a FE model to investigate the kinematics and contact pressures of the BOX^®^. However, to our knowledge, no FE study has investigated TAR implant-bone micromotion.

Manufacturers of TAR prostheses provide detailed guidelines for their positioning during arthroplasty surgery. Proper implant positioning is necessary for achieving good clinical results [24], [25], and even a slight degree of malpositioning has been claimed to result in higher failure rates [26]. Malpositioning of TAR has also been investigated in biomechanical settings. Saltzman et al. [24] found that elongation of the tibiocalcaneal ligament was considerably increased by varus/valgus malpositioning, and Espinosa et al. [22] found that such malpositioning increased pressures acting on the mobile component, which could lead to premature PE wear. Varus/valgus and dorsi-/plantar-flexed malpositioning of TAR components reported in the literature [27] may lead to a gap between the implant and the bone (often seen clinically on post-operative x-rays), which is likely to result in increased micromotion (as identified in a study assessing micromotion of a prosthetic glenoid, [16]). However, despite these clinical observations, the impact of TAR malpositioning on implant primary stability remains unexplored.

The aim of this study is to use *in silico* modelling to calculate implant-bone micromotion and peri-implant bone strains of the tibial and talar components of current TAR designs when optimally positioned and malpositioned. These data will identify fixation features and positioning scenarios that place the ankle prosthesis at higher risk of early loosening. The findings can be useful for surgeons and implant designers when planning the arthroplasty procedure.

## 2 Methods

### 2.1 Geometrical modelling

**Fig. 1:**
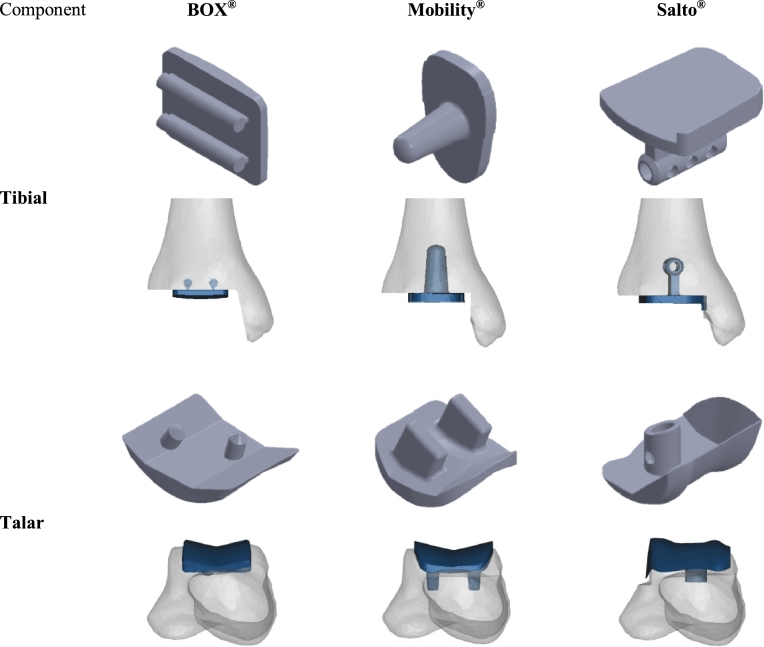
Geometrical computer-aided-design models of the tibial and talar components of the three total-ankle-replacement (TAR) prostheses explored in the study; the positioning of each design with respect to the bone is also shown from a frontal view. Both BOX^®^ components achieve fixation to the bone closest to the joint line (fixation features anchored to the dense distal tibial or proximal talar bone) and via two fixation pegs. The tibial Mobility^®^ and talar Salto^®^ components achieve fixation to the bone furthest from the joint line (fixation features extended deeper into the less dense trabecular bone) and via a single peg each. The Salto^®^ talar component has a flange that covers the lateral facet of the talus (after bone resection).

The geometries of the BOX^®^ (MatOrtho, Leatherhead, UK), Mobility^®^ (DePuy, Warsaw, IN, USA) and Salto^®^ (Tornier, Amsterdam, The Netherlands) TAR designs – which have been three of the most commonly implanted TAR devices in the 2010s according to national joint replacement registries [3], [4], [5], [6], [7] – were reverse-engineered from production specimens using a digital Vernier Calliper, micrometer and digital photography by means of computer-aided-design software (SolidWorks^®^, Education Edition, 2011–12; Dassault Systèmes, France) (Fig. 1).

A cadaveric leg cut below the knee joint (female, age 79 years, height 170 cm, body mass 59 kg, no known bone or leg anatomical abnormalities) was CT-scanned in a ‘neutral’ position (approximately 90° between the posterior calf and the sole of the foot) using a Definition AS^®^ Computed Tomography (CT) scanner (Siemens Healthcare, Erlangen, Germany); axial voxel sizes were set to approximately 0.56 mm and slice thicknesses were 0.6 mm. Geometrical models of the tibia and talus were then generated using MIMICS^®^ (version 16.0; Materialise NV, Leuven, Belgium).

**Fig. 2:**
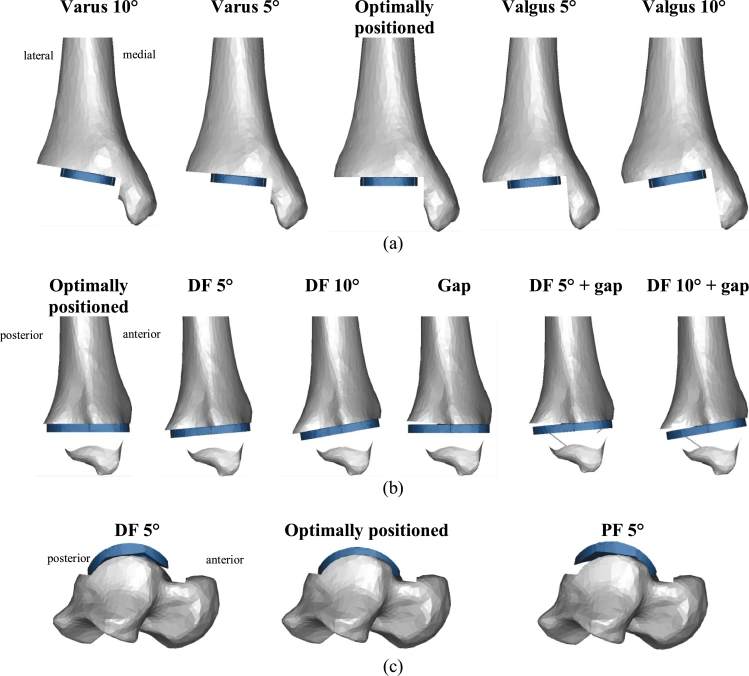
Geometrical models simulating tibial- and talar-component ‘optimal’ positioning and malpositioning (Mobility as an example): (a) the tibial optimally positioned and varus/valgus-malpositioned (5° and 10°) model variants from a frontal view; (b) the tibial optimally positioned, dorsiflexed-malpositioned (5° and 10° of DF) and DF combined with a posterior-gap-malpositioning model variants from a lateral view; (c) the talar optimally positioned, dorsiflexed-malpositioned (5° of DF) and plantarflexed-malpositioned (5° of PF) model variants from a lateral view.

Implant sizes were rescaled according to the subject's anatomy. Virtual implantations were performed in Rhinoceros^®^ (version 4.0; Robert McNeel & Associates, Seattle, WA, USA) according to the surgical guidelines provided by the prosthesis manufacturers [28], [29], [30], [31]. Briefly, the surgical technique requires the distal tibia to be cut in the anteroposterior direction with a drill and/or sagittal saw, using a designated instrument to align the cuts appropriately. The talar surface is then exposed by plantarflexing the foot, and holes for pegs are drilled in the superoinferior direction. In addition to the ‘optimal’ position, several types of malpositioning were simulated, including varus/valgus and dorsiflexed positioning of the tibial component, as well as implant positioning with and without a 1–2 mm gap between the tibia/talus and implant component (Fig. 2). These represent the most common and worrying types of TAR malpositioning, as determined from the literature [22], [24], [26], [27], [32], [33] and through consultation with an orthopaedic surgeon specialised in foot and ankle surgery (JC), who supervised the ‘virtual implantation’ process.

### 2.2 Material properties

Implants were assigned a Young's modulus of 210 GPa and Poisson's ratio of 0.3 to represent CoCr alloy. Bone was assigned a Poisson's ratio of 0.3, and each element of the FE model was assigned an individual elastic modulus that depended on the average CT greyscale value (in Hounsfield Units, HU) of all voxels contained within the element volume according to equations derived in previous studies [34], [35], [36], [37], as described in the following empirical equations:

(1) $$\rho =0.0405+\left(9.18\times 10^{-4}\right)\;HU$$

**Equation 1**: An empirical relationship between bone density (g/cm^3^) and radiographic greyscale (Hounsfield Units, *HU*) used in this study, as derived from previous studies [34], [38].

where *ρ* is the apparent dry density (g/cm^3^) of the bone area of interest and *HU* is the radiographic greyscale of this area in *HU*.

(2) $$E=\;\left\{ \begin{array}{c} \begin{array}{c} 3.60\rho -0.14\;0<\rho \leq 0.1\;\\ 18.49\;\rho^{1.93}\;1<\rho \leq 0.37 \end{array}\\ 8.87\rho -0.57\;0.37<\rho \leq 1.5\\ 4.83\;\rho^{2.39}\;\rho >1.5 \end{array}\right.$$

**Fig. 3:**
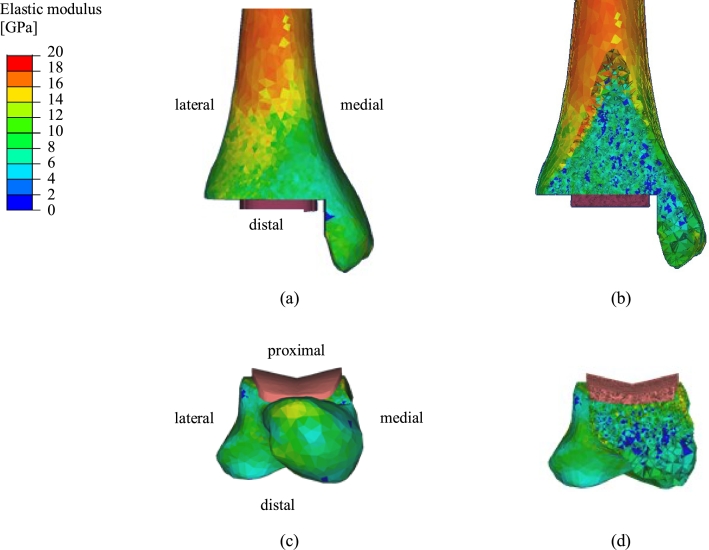
Image representations of the elastic moduli assigned to elements forming the bone surface ((a), (c)) and bulk ((b), (d)) as produced in MIMICS^®^. Elastic moduli of elements containing cortical bone (i.e. those on the bone surface) were larger than those of elements consisting of purely trabecular bone (i.e. those within the bone bulk). Elastic moduli of elements containing tibial cortical bone further from the implant (i.e. tibial diaphysis) were particularly large. (For interpretation of the references to colour in this figure legend, the reader is referred to the web version of this article.)

**Equation 2**: Elastic moduli (GPa) assigned to the tibia and talus based on empirical relationships derived from previous studies [35], [36], [37] and linear interpolations.

where *E* (GPa) is the elastic modulus assigned to the element and *ρ* (g/cm^3^) is the dry apparent density of bone volume contained within the element. Images demonstrating the elastic moduli assigned to elements forming the bone are shown in Fig. 3.

### 2.3 Contact

Linear isotropic Coulomb friction (0.5 coefficient of friction, CoF) was assumed at the bone-implant interface in line with previous literature [39]. Friction coefficient was also set to 0.4 and 0.6 in the framework of a sensitivity analysis, which was shown to affect the model outcomes to a minor extent (7% and 1% mean difference for the micromotion and strain outcomes specified below, respectively). ‘Hard’ linear contact model with penalty method and automatically calculated contact stiffness were used to simulate the normal behaviour at the bone-implant interface; small-sliding formulation and surface-to-surface discretisation method with minimal tolerance were used at the interface to reduce the likelihood of penetration. These were in line with research in the field of FEM in orthopaedic applications [40], [41].

### 2.4 Boundary conditions

**Fig. 4:**
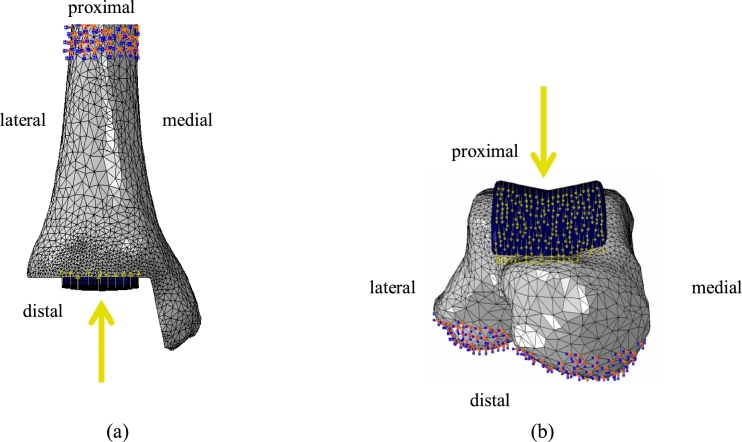
Anterior view demonstrating the geometry of tibial (a) and talar (b) model variants (the BOX design is shown as an example) as produced in ABAQUS^®^. The tibia and talus are shown in grey, while the device components are shown navy blue. The orange/blue dots indicate fixations against all three degrees of freedom; the yellow arrows pointing vertically indicate the force applied to the nodes comprising the articular surfaces of the tibial and talar components. (For interpretation of the references to colour in this figure legend, the reader is referred to the web version of this article.)

The proximal three quarters (by length) of the tibia and distal quarter of the talus were fixed to all motions (Fig. 4). These were found to be acceptable in a preliminary sensitivity study applied to all ‘baseline’ model variants (12 in total; all optimally positioned), in which boundary conditions (BCs) were also set to fix shorter segments of the bones (the proximal quarter of the tibia anddistal tenth of the talus). The pass criterion of the sensitivity study was when the influence of BCs on the strain and micromotionoutcome measures was smaller than 10%, or when the differences in mean strain and micromotion were smaller than 0.1% or 10 µm, respectively.

### 2.5 Loading conditions

Loading conditions (LCs), applied as point forces evenly distributed between the nodes forming the implant articular surfaces (Fig. 4), simulated the physiological peak axial contact load acting on the implant tibial and talar components during gait. This was estimated in a previous study [42] to occur at 45–50% gait-cycle (GC) (terminal stance, just after heel rise), and to equal 5.2-times bodyweight. A preliminary study in which the ankle contact forces acting in six segments of the stance phase of gait (2%, 12%, 31%, 45%, 50%, 55% GC) were applied to the model, confirmed that the implant-bone micromotion and bone strains were the largest at 45–50% GC, and accordingly, this was selected as the segment of stance phase to be simulated in the current model. Shear forces were not considered as these are small [43], [44], [45], and should not be normally transmitted to the implant fixation interface due to the floating nature of the PE bearing of the implants considered. To investigate the effect of shear forces possibly occurring at the interface between the PE and metal components of the implant on the model outcome measures (see below), we conducted a preliminary sensitivity study in which small shear forces (acting posteriorly and of magnitude equal to the product of the axial compression force and the estimated PE-CoCr CoF, 0.07; [46]) were applied to all ‘baseline’ model variants (24 in total) in addition to the aforementioned compressive load. Such shear was found to affect outcome measures only slightly: tibial micromotion outcomes were affected by less than 10% (4% on average), while strain outcomes were affected by up to 3% (1% on average); talar micromotion outcomes were affected by less than 3% (1% on average), while strain outcomes were affected by 1% on average.

Since all implant designs considered have mobile PE bearings, the point of load application is not certain. Three loading cases were therefore implemented to represent a centrally, anteriorly and posteriorly located PE bearing by applying the loading to centrally, anteriorly and posteriorly located halves of the articular implant surfaces, respectively, so to cover the positions of the PE component during stance [20], [22], [23].

### 2.6 Meshing

Automatic meshing was applied in 3-matic^®^ (version 16.0; Materialise NV, Leuven, Belgium) using solid linear tetrahedral elements (of type C3D4 in ABAQUS; mesh density of maximum triangle edge length 5 mm); finer meshing (maximum triangle edge length 1.5 mm) was applied at the vicinity of the bone-implant interface. The meshes of the tibial and talar modelvariants comprised 32,000–48,000 and 21,000–29,000 3-degrees-of-freedom nodes, respectively. These meshes were consideredacceptable based on a mesh-refinement study in which three different mesh densities (of 3.0-, 2.0- and 1.5-mm edge length at the vicinity of the bone-implant interface) were implemented to each of the ‘baseline’ model variants (24 in total; all optimally positioned, while applying four different loading scenarios). This preliminary investigation into mesh convergence demonstrated that differences in outcome measures between the 2.0-mm- and1.5-mm-edge-length meshes were considerably smaller compared with the differences between the 3.0-mm- and 1.5-mm-edge-length meshes. For the tibial component model variants, for example, the average difference in mean and peak tibial micromotion (percentagewise) between the 3-mm- and 1.5-mm-edge-length meshes was 8% and 9%, respectively, but this reduced to 4% and 5% for the 2-mm-edge-length mesh. A similar trend was observed for the talar micromotion, as well as for bone strains. Meshes with characteristic edge length of 1.5 mm, which were the finest meshes we were able to utilise given the complexity of the model, the number of model variants and the computational resources we had available, were thus consistently implemented. It should be noted that meshes of equivalent element type and similar density were used and experimentally validated in previous studies conducted in our group to assess micromotion of a glenoid implant inserted into a porcine scapula [16], and strains occurring at the vicinity of atibial component used in knee replacement inserted into a cadaveric human tibia [47].

### 2.7 Numerical method

The ABAQUS^®^ Standard/Implicit FE solver (ABAQUS CAE, ver. 6.11-2, SIMULIA, Providence, RI, USA) in its nonlinear analysis mode was used to process all 117 model variants and 102 preliminary model variants. The runtime of each tibial and talar model variant was approximately 1 h and 30 min, respectively, when using a 64-bit Microsoft Windows 7-based desktop computer with a CPU comprising Intel Core i7 3.4 GHz 4-cores and 8 GB RAM.

### 2.8 Outcome measures

A script was coded in MATLAB^®^ (version R2013a, MathWorks Inc., Natick, MA, USA) to calculate the distribution of total, normal and tangential micromotion at the bone-implant interface and determine peak (95th percentile) and mean micromotion for each model variant. The bone-implant interface area subjected to total micromotion larger than 100 µm [48] was calculated; this is the mid-range of the 50–150 µm range identified in previous studies as containing a critical micromotion level above which osseointegration is less likely to occur [8], [9], [10]. Additionally, distributions of maximum and minimum principal strains within the bone were calculated, and peak and mean values of these within a bone volume defined by an offset surface of 10-mm from the bone-implant interface (region of interest, ROI) were determined. A custom script was also used to calculate the volume of bone exposed to strains larger than yield strains, expressed as the ratio of this volume over the total volume of all elements in the ROI. The bone yield strains were 0.73% (compressive) and 0.65% (tensile) [49].

## 3 Results

### 3.1 Optimally positioned implants

#### 3.1.1 Tibial components

**Fig. 5:**
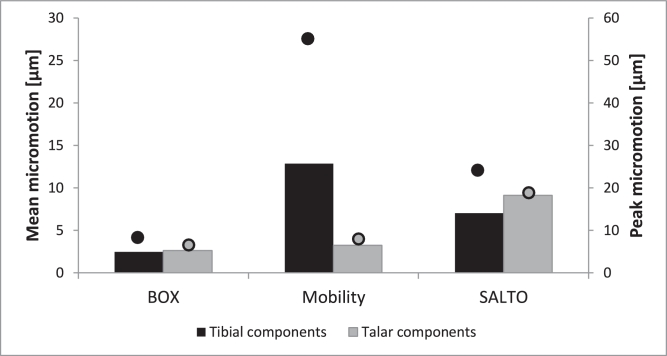
Micromotion occurring at the interface between the bone (tibia/talus) and implant component at 45% gait-cycle (GC) for all TAR designs (BOX, Mobility and Salto) considered in this study (only centred-loading model variants are shown here). Outcomes shown include mean (bars) and peak (dots) implant-bone micromotion (in µm) for each model variant. Micromotion did not exceed the threshold value of 100 µm.

**Fig. 6:**
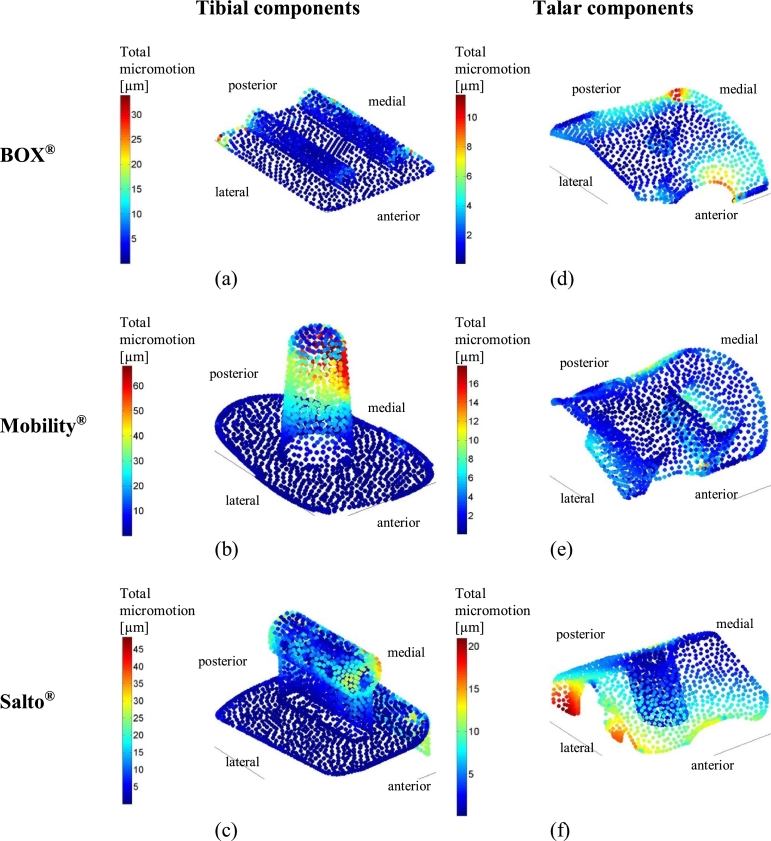
Total micromotion occurring at the interface between the bone (tibia/talus) and implant component at 45% GC for all TAR designs (BOX ((a), (d)), Mobility ((b), (e)) and Salto ((c), (f))) considered in this study (only centred-loading model variants are shown here). (For interpretation of the references to colour in this figure legend, the reader is referred to the web version of this article.)

BOX and Salto demonstrated smaller tibial micromotion than Mobility (Fig. 5). This was found regardless of whether the PE component was located centrally, posteriorly or anteriorly. The peak micromotion in Mobility was at the proximal, posteromedial side of the fixation stem (Fig. 6b), with tangential movement being considerably larger than normal movement. The peak micromotion of Salto was at the anterior and posterior of the proximal keel, and also along the side flange where the implant interfaced the sagittal cut plane of the tibia (Fig. 6c). The largest micromotion of the BOX tibial component was on the posterior of the cylindrical keels and the flat fixation surface (Fig. 6a), with roughly equal tangential and normal movements.

**Fig. 7:**
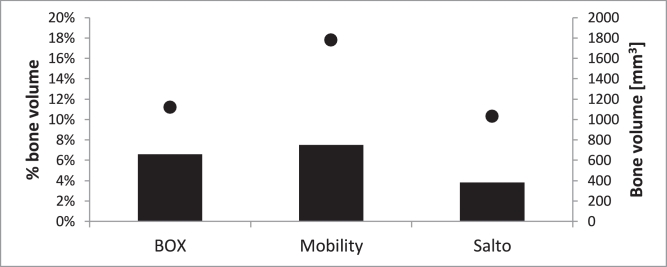
% bone volume (bars) and bone volume in mm^3^ (dots) contained in the tibial region of interest (1-cm distance from the bone-implant interface) subjected to strains larger than yield strain (0.73% and 0.65% compressive and tensile strains, respectively), for all TAR designs (BOX, Mobility and Salto) considered in this study at 45% GC (only centred-loading model variants are shown here).

The peak and mean strains in the tibia were similar for the three implant designs (mean and peak maximum principal strains: 0.13–0.15% and 0.37–0.45%; mean and peak minimum principal strains: 0.25–0.32% and 0.68–0.89%). Specifically, maximum and minimum principal strains in the distal tibial cortex were predicted to be approximately 0.25% and 0.30%, respectively, for all implant designs. Despite the similar levels of strain, Mobility had ∼1800 mm^3^ (8%) of bone contained in the ROI subjected to above-yield strains, compared with ∼1100 mm^3^ (7%) for BOX and ∼1000 mm^3^ (4%) for Salto (Fig. 7). In all designs, peak strains were recorded around the implant fixation features and the perimeter of the flat face of the implant.

#### 3.1.2 Talar components

Implant-bone micromotion was generally smaller in the talus than in the tibia. Salto demonstrated the largest micromotion(Fig. 5). Micromotion peaked at the anterior and posteromedial edges of the talar component for BOX, along the medial peg and at the posterior edge for Mobility, and at the lateral implant-talus interface (flange) for Salto (Fig. 6).

Talar trabecular bone strains were similar for all implant designs, though approximately half the magnitude calculated for the tibia (mean maximum principal strains: 0.06–0.07%, mean minimum principal strains: 0.16–0.18%). The volume of bone contained in the ROI subjected to above-yield strains was ∼350 mm^3^ (1.3%) for the Mobility, ∼200 mm^3^ (0.8%) for the Salto and ∼150 mm^3^ (0.5%) for the BOX talar components. Strains in the bone ROI peaked around the centre of the talus for all designs, which is where the average trabecular bone density was lower.

### 3.2 Malpositioned implants

#### 3.2.1 Tibial malpositioning: varus/valgus

Varus/valgus malpositioning of the tibial component affected implant-bone micromotion and peri-implant bone strains to a much lesser extent than if a gap was present between the bone and prosthesis (see below); for all implant designs subjected to the centred loading case, mean changes in micromotion and principal strain outcomes with respect to the equivalent optimally positioned case were within the ranges of 0–22% and 0–16%, respectively.

#### 3.2.2 Tibial malpositioning: dorsiflexion (no posterior gap)

Dorsiflexion of the tibial component affected implant-bone micromotion and peri-implant strains to a much lesser extent than a gap between the bone and the implant; for all implant designs subjected to the centred loading case, mean changes in micromotion and principal strain outcomes with respect to the optimally positioned case were within the ranges of 3–16% and 0–10%, respectively.

#### 3.2.3 Tibial malpositioning: posterior gap between the tibia and tibial component

**Fig. 8:**
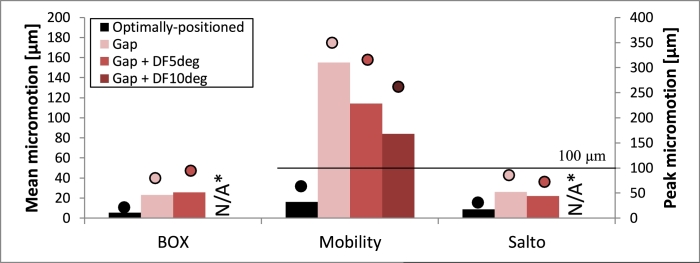
Micromotion occurring at the tibia–tibial component interface at 45% GC for the optimally positioned and all posterior-gap-malpositioned cases (with/without dorsiflexed malpositioning, DF), and all TAR designs (BOX, Mobility and Salto) considered in this study. Only the posterior-loading model variants – for which the increase in implant-bone micromotion with respect to the optimally positioned model variants was the greatest – are shown here. Outcomes shown include mean (bars) and peak (dots) implant-bone micromotion. A horizontal line highlights cases in which micromotion exceeded the threshold value of 100 µm. * Two bars are missing due to technical difficulties in producing the models.

**Fig. 9:**
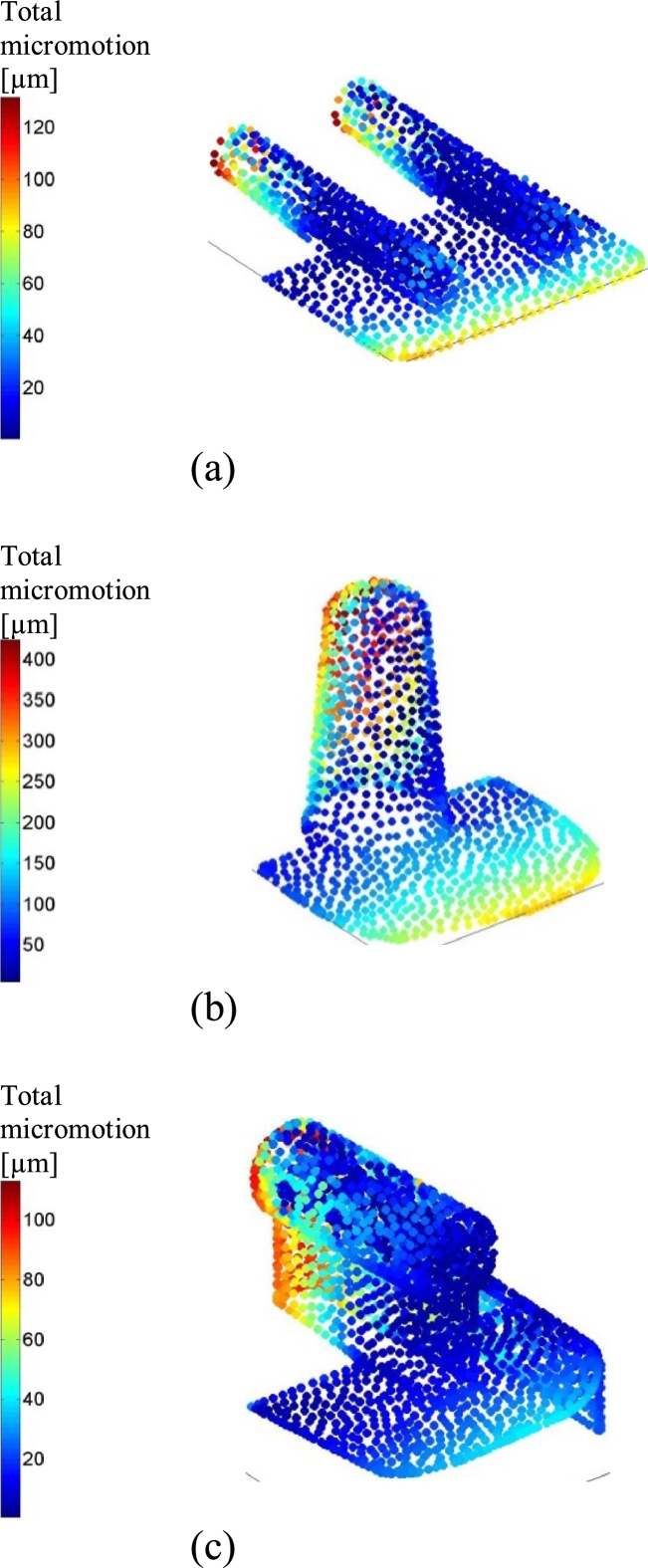
Total micromotion occurring at the tibia–tibial component interface at 45% GC for the posterior-gap-malpositioned (no dorsiflexion) TAR designs (BOX (a), Mobility (b) and Salto (c)) considered in this study. Only the posterior-loading model variants are shown here. (For interpretation of the references to colour in this figure legend, the reader is referred to the web version of this article.)

A gap at the posterior part of the tibial fixation surface caused a considerable increase in micromotion for all designs (with respect to the optimally positioned case), and this increase was largest when the PE component was located posteriorly (Fig. 8). Mobility showed the largest increase, with peak micromotion increasing up to approximately 5.5-fold to exceed the threshold value of 100 µm. For the BOX and Salto tibial components the posterior gap resulted in a similar increase (percentagewise) in implant-bone micromotion, although the magnitudes were far less (Fig. 8). For all designs, the increased micromotion was at the anterior of the tray (mainly normal movement) and posterior of the fixation keel(s) (mainly tangential movement; Fig. 9).

Peri-implant tibial strains were also elevated by posterior-gap malpositioning. Particularly, ROI bone volumes subjected to above-yield strains increased from 11% to up to 29% for the Mobility design (posterior loading); the increase was slightly smaller for BOX (from 13% to 23%) and smallest for Salto (from 7% to up to 9%). Distributions of tibial strains around the posterior-gap-malpositioned implant were similar to those of the optimally positioned model variants (Section 3.1.1).

#### 3.2.4 Talar malpositioning: dorsiflexed/plantarflexed implantation creating anterior/posterior gap between the bone and implant

**Fig. 10:**
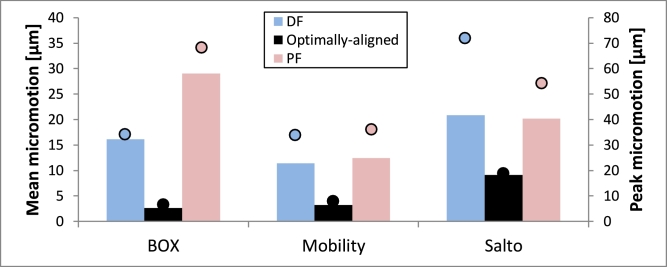
Micromotion occurring at the talus–talar component interface at 45% GC for the optimally positioned and dorsi-/plantar-flexed- malpositioned cases, and all TAR designs (BOX, Mobility and Salto) considered in this study (only centred-loading model variants are shown here). Outcomes shown include mean (bars) and peak (dots) implant-bone micromotion. DF – dorsiflexed malpositioning, PF – plantarflexed malpositioning.

Dorsi-/plantar-flexed malpositioning of the talar component resulted in a considerable increase in implant-bone micromotion (but for all designs, values remained below the 100-µm threshold; Fig. 10). Mobility demonstrated the smallest micromotion under these conditions. Anterior and posterior loading further increased micromotion outcomes for the dorsiflexed and plantarflexed malpositioned cases, respectively.

Peri-implant talar strains were also increased by dorsi-/plantar-flexed malpositioning of the talar component. Bone volumes subjected to above-yield strains, for example, increased from 1% to up to 7% for the malpositioned Salto, from <1% to up to 4% for BOX and from >1% to 2% for Mobility with centred loading. Anterior and posterior loading affected the dorsiflexed and plantarflexed malpositioned cases to a greater extent. Talar strains at the dorsi-/plantar-flexed-malpositioned implant proximity peaked in regions similar to those in which they peaked for the optimally positioned model variants (Section 3.1.2).

## 4 Discussion

### 4.1 Key findings

Qualitative interpretation of the results of this study demonstrates that implant-bone micromotion and bone strains in TAR are strongly influenced by the implant design and positioning. Reduced micromotion was predicted for the component designs which relied on more than a single peg to achieve fixation and had their fixation features anchored to the dense distal tibial or proximal talar bone (BOX tibial and talar components), compared with those with a single fixation peg extended deeper into the less dense trabecular bone (Mobility tibial and Salto talar implants; Fig. 1). However, the most dominant influence on bone strains and micromotion, more so than implant design, was when a gap was present at the fixation surface of the prosthetic device. This elevated strains and micromotion in all designs, and the data indicate this should be avoided, even at the expense of other types of implant malpositioning. Of all tibial components, the Mobility design was most affected by such malpositioning, which culminated in micromotion values exceeding the 100-µm threshold. The Mobility talar component, however, was least affected by gap-related malpositioning, and none of the three talar components was subjected to micromotion larger than 100 µm. When forces were applied to the part of the implant not fully seated on the bone, the increase in implant-bone micromotion and bone strains was particularly large, which can be attributed to the ‘rocking horse’ mechanism [18], [26].

### 4.2 Comparison between implant designs when optimally positioned

Our data indicate that the optimally positioned Mobility tibial component may be more prone to loosening than the other designs. It is possible that this is due to the single, long conical stem method of achieving fixation, which transmits loads deep into the lower-stiffness trabecular bone [50]. The single post may also offer less stability against rotation about its own axis than the other designs [50], which is corroborated by the micromotion being dominated by sliding. The two bars on the BOX tibial component, as well as the anteroposterior bar on the Salto, provided more stability. These findings are in line with slightly higher revision rates reported for Mobility compared with BOX and Salto [4], [6]. It is acknowledged, however, that the BOX and Salto are relatively new in clinical use and thus lack detailed data of survival rates; care should therefore be taken when drawing such conclusions.

For the talar component, Salto had the largest micromotion outputs. This may be explained by the fact that the implantation of BOX and Mobility preserves more of the cortical sidewalls of the talus, which helps to maximise the bone support of the implant [51]. The Mobility and BOX components possibly provide more favourable conditions for bone ingrowth because they achieve fixation via features closest to the joint line, where bone is normally stiffer, and also rely on more than a single fixation peg, which is likely to contribute to the stability at the bone-implant interface.

The higher micromotion and strain outputs recorded for the tibial compared with the talar components correspond well with clinical observations. Previous research relying on radiostereometric analysis to detect implant instability of Mobility found that the tibial component was more prone to load-induced displacements [50], [52], implying larger early implant-bone micromotion. Other radiograph-based studies reported larger lucencies indicative of implant loosening for the tibial compared with the talar component for Mobility [53] and Salto [54]. This may be attributed not only to the component design, but also to the fact that the tibial component, more so than the talar implant, rests almost entirely on trabecular bone, making it more likely to migrate [51]. Data of some national registries [4], [5], [55] corroborate these findings by reporting higher loosening-caused revision rates for the tibial component. However, numbers reported remain small and care should be taken with their interpretation.

Strains occurring on the surface of the distal tibia agreed with data of tibial cortical strains reported in previous research for typical activities (reviewed in [56], [57]). The current findings indicating that tibial strains were particularly elevated around the cylindrical part of the keel and immediately to the distal tray of the Salto design are supported by the findings of a previous study employing experimental and computational modelling [19], [20].

### 4.3 Tibial malpositioning: varus/valgus and dorsiflexion

Micromotion and strain outcomes did not increase due to varus/valgus or dorsiflexed positioning of the tibial component. This is partially in line with a recent clinical study concluding that the effects of mild coronal or sagittal malpositioning of the Hintegra^®^ TAR on midterm (mean: 4 years) clinical outcomes were statistically insignificant [58]. Also, a FE study exploring the effects of glenoid component inclination on implant-bone micromotion reached similar conclusions [17]. However, such malpositioning should be avoided as it has been shown to increase ligament elongations [24] and contact pressures acting on the mobile component [22].

### 4.4 Tibial/talar malpositioning: gap between the bone and implant

Situations in which the implant component was not fully seated on the bone resulted in the largest increase in bone strains and implant-bone micromotion. The Salto and BOX tibial designs were less sensitive to gap malpositioning, which is attributable to the non-constant axial cross section of their fixation keels. Mobility demonstrated the greatest increase, manifested in elevated sliding of the posterior part of the fixation stem, which is possibly a result of the compromised rotational stability provided by the stem and the depth of the fixation surface, anchored to the less dense trabecular bone, more proximally with respect to the joint line. For the talar component, Mobility was the least sensitive design for such implant malpositioning, which is possibly attributed to the double keel design fixation, with bigger fixation features than the BOX. The importance of a gap at the bone-implant interface was previously reported [16], where a gap of only 135 µm between the scapula and glenoid component investigated, resulted in peak micromotion increasing from 80 to 180 µm, which is comparable to the increase found in this study.

### 4.5 Limitations

The main limitation of the model is manifested in the fact that the computationally obtained results were not directly validated experimentally. However, the model generation protocol was the same as in experimentally validated FE models of knee and shoulder replacements developed in our group [16], [47]. The agreement between our findings and those derived from previous studies (experimentally and computationally based), and the evidence from clinical studies supporting the findings, can demonstrate the value of the current work. We also limited our interpretation to qualitative comparison, and that has yielded useful comparative findings for the different implant designs and positioning variations. Additionally, only a single subject was used to construct the FE models despite the fact that bone peak strains and stresses predicted utilising FE modelling, have been previously shown to be influenced by inter-subject variability reflected in bone quality and stiffness distribution (e.g. a FE model of the intact acetabulum, [59]). Yet, the conclusions regarding the effects of TAR design and positioning on the stability of the device and success of treatment – which were drawn from a comparative, qualitative interpretation of the model-predicted bone strains and implant-bone micromotion – are not expected to change by extending the study to more subjects. Also, the model developed in this study focussed on a single segment of the GC. Nevertheless, the segment of the GC simulated here (45–50%) was found to be the one in which the force applied to the TAR components – as well as the implant-bone micromotion and bone strains – peak; accordingly, it can be considered as the ‘worst case scenario’ in terms of implant primary stability. Cases in which the mobile PE insert transferred not only compressive loads but also shear forces to the implant bearing surfaces were modelled as well, and the numerous loading cases implemented covered several possible positions of the PE bearing during gait. All FE models are limited by the assumptions made at the bone-implant interface, where touching surfaces are modelled in perfect contact with a uniform CoF. In reality, however, the interface is made of two rough surfaces that are not necessarily in perfect contact, and indeed the CoF may vary depending on bone density. To elucidate this, we performed a sensitivity study and found that the CoF had a marginal impact on the calculated strain and micromotion outputs, which may have been due to the loading configurations of the implants in this particular study and should not be generalised to all implant-bone interactions. Another limitation of the current model is that, similarly to many studies exploring joint replacement devices, it did not consider the initial loads acting at the bone-implant interface as a result of press-fit. Nevertheless, Viceconti et al. [41] found that a press-fitted frictional contact model demonstrated only minor benefit over a purely frictional model in predicting implant-bone micromotion when the CoF was larger than 0.3 (here 0.5). Finally, the model did not consider bone ingrowth stabilising implant fixation or possible bone deposition bridging gaps between the bone and the malpositioned implant component. The reason for this is that the model was designed only to explore implant-bone micromotion of TAR designs at ‘time-zero’ – as indicative of implant primary stability and potential of osseointegration-induced fixation [12]. Considering these and other limitations in the modelling, it is recommended that the data presented in this study be interpreted mostly as trends of effects, rather than as absolute values.

## 5 Conclusions and clinical relevance

The work described herein is the first to use FE modelling to investigate implant-bone micromotion of TAR prostheses; it is also the first work to compare some of the most commonly used TAR designs when subjected to physiological loading in terms of implant-bone micromotion and peri-implant bone strains.

The model presented here provides a useful tool to clinicians and implant designers. Fixation close to the joint line while preserving more of the cortical sidewalls of the bone and/or relying on more than a single peg was found to be beneficial. Fixation features spread over a large area of the implant tray and bone resection surface (large anteroposterior and/or mediolateral length), as well as a non-constant axial cross section, can further enhance the stability of the prosthesis against internal/external rotation and coronal/sagittal tilting. More important than implant design, however, is the surgical technique to ensure both implant component fixation surfaces are fully seated on the bones to minimise the risk of excessive early micromotion, loosening and failure of the arthroplasty. Accordingly, there may be a greater opportunity to improve TAR outcomes through surgical training and instrument design rather than new implant designs.

## Conflict of interests

The authors have no conflicts of interest.
